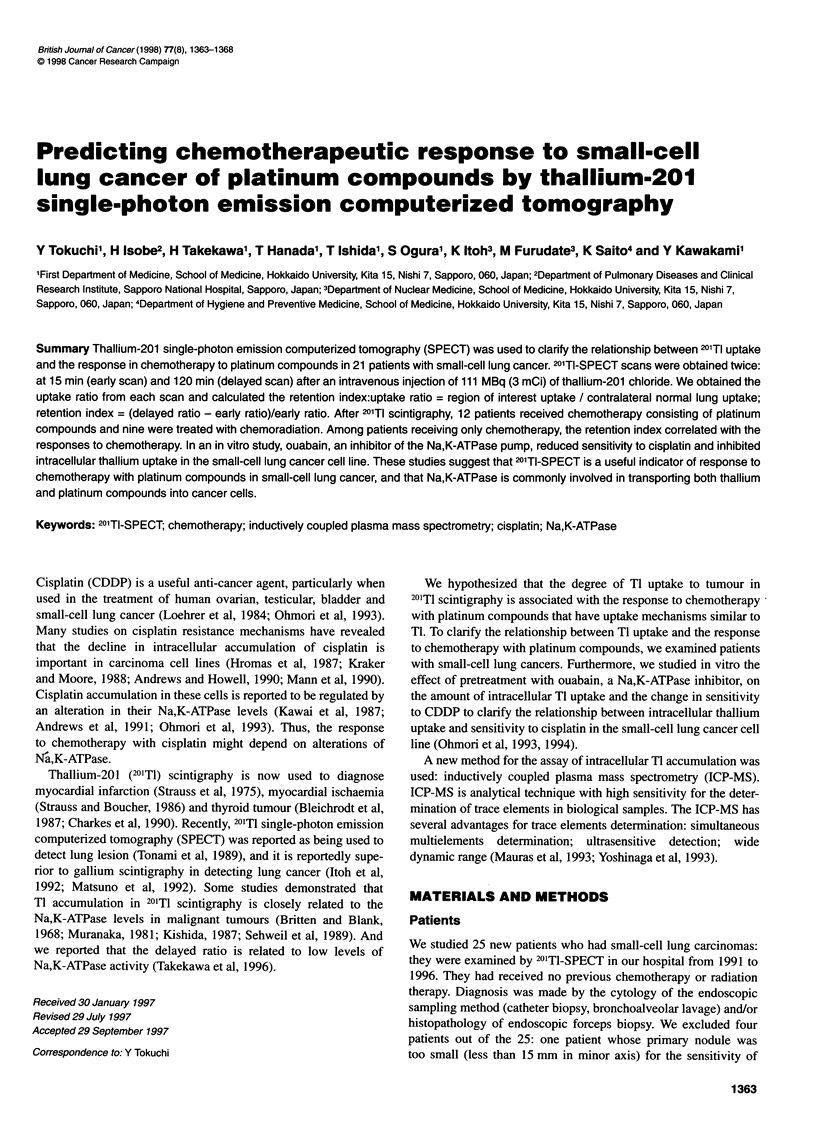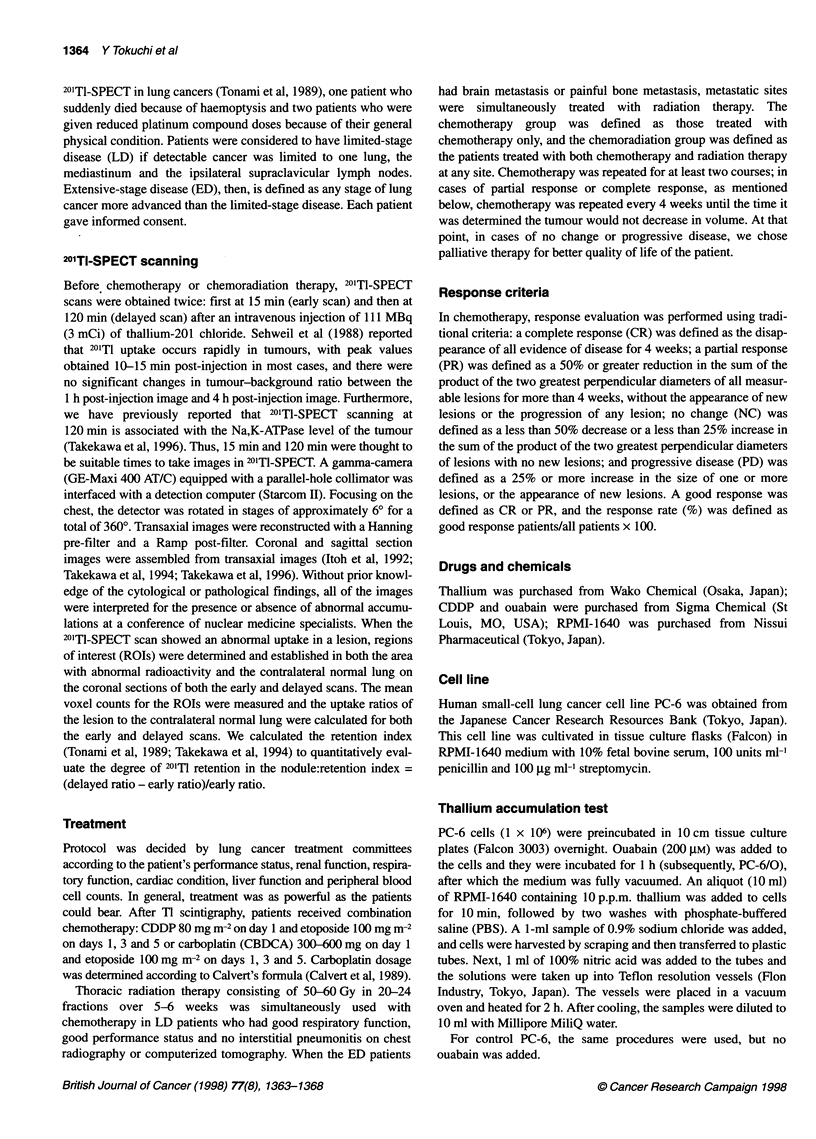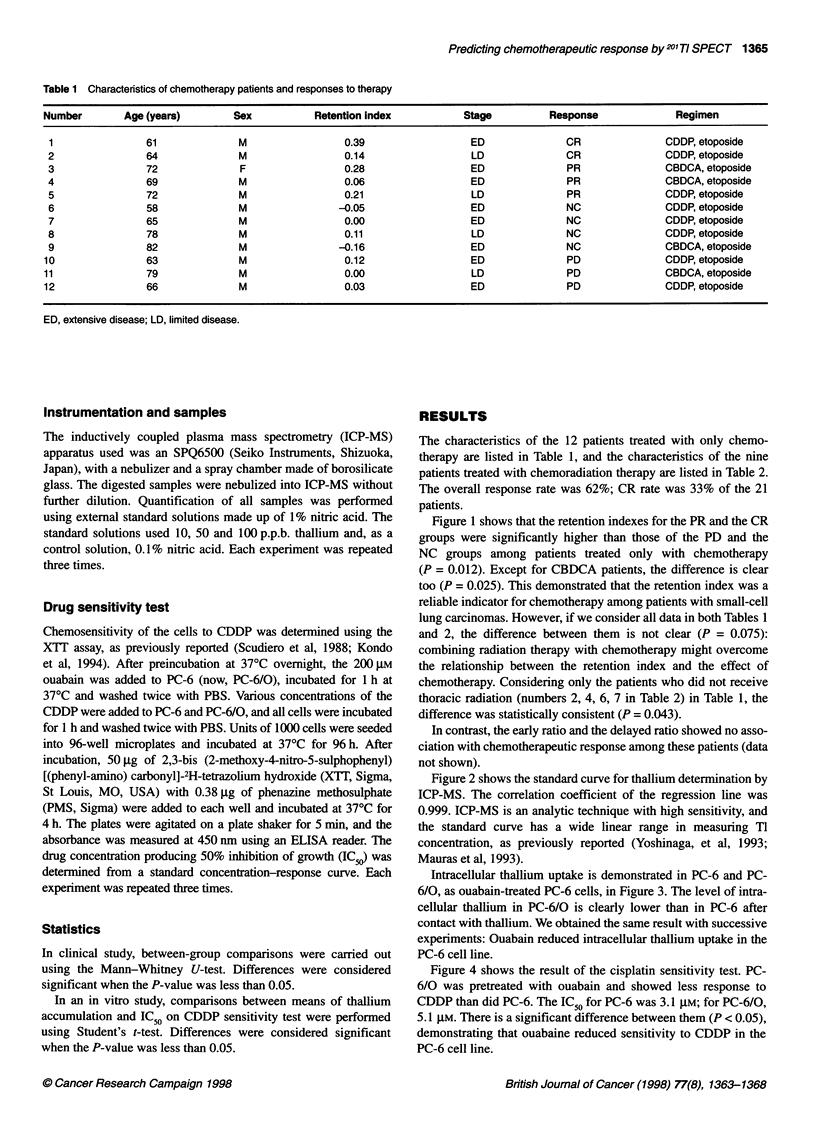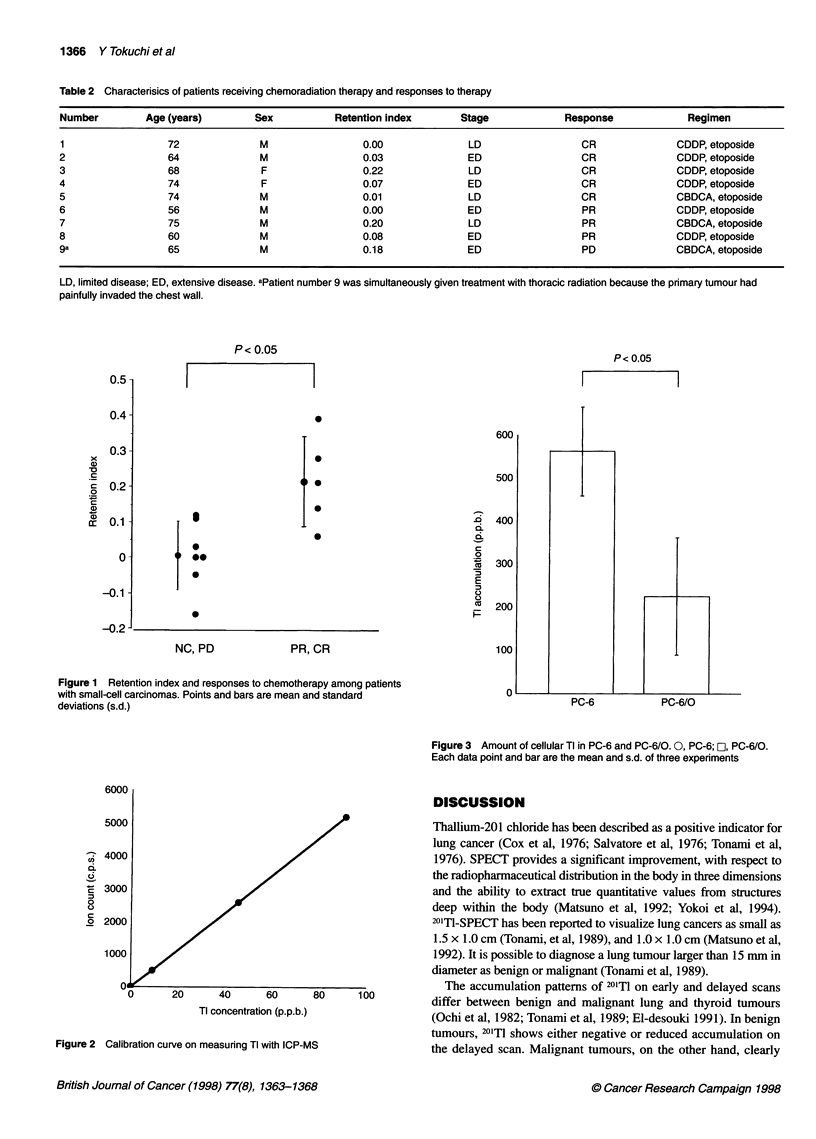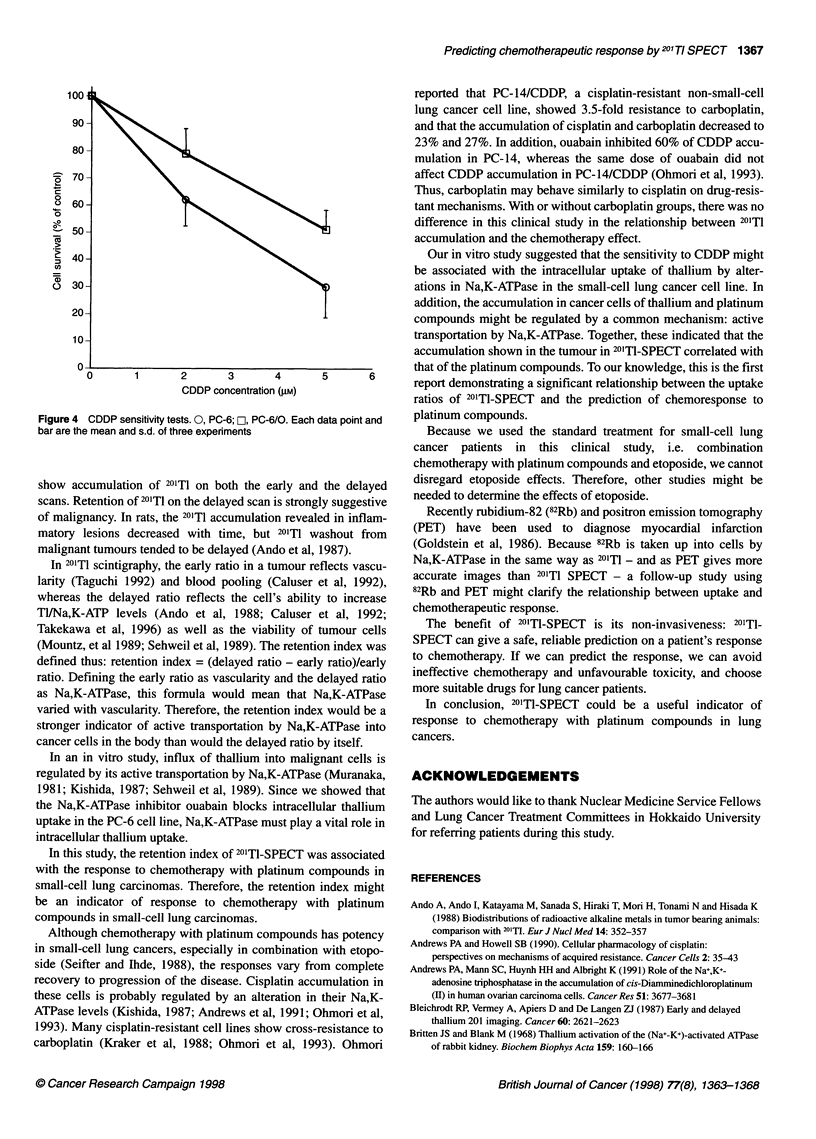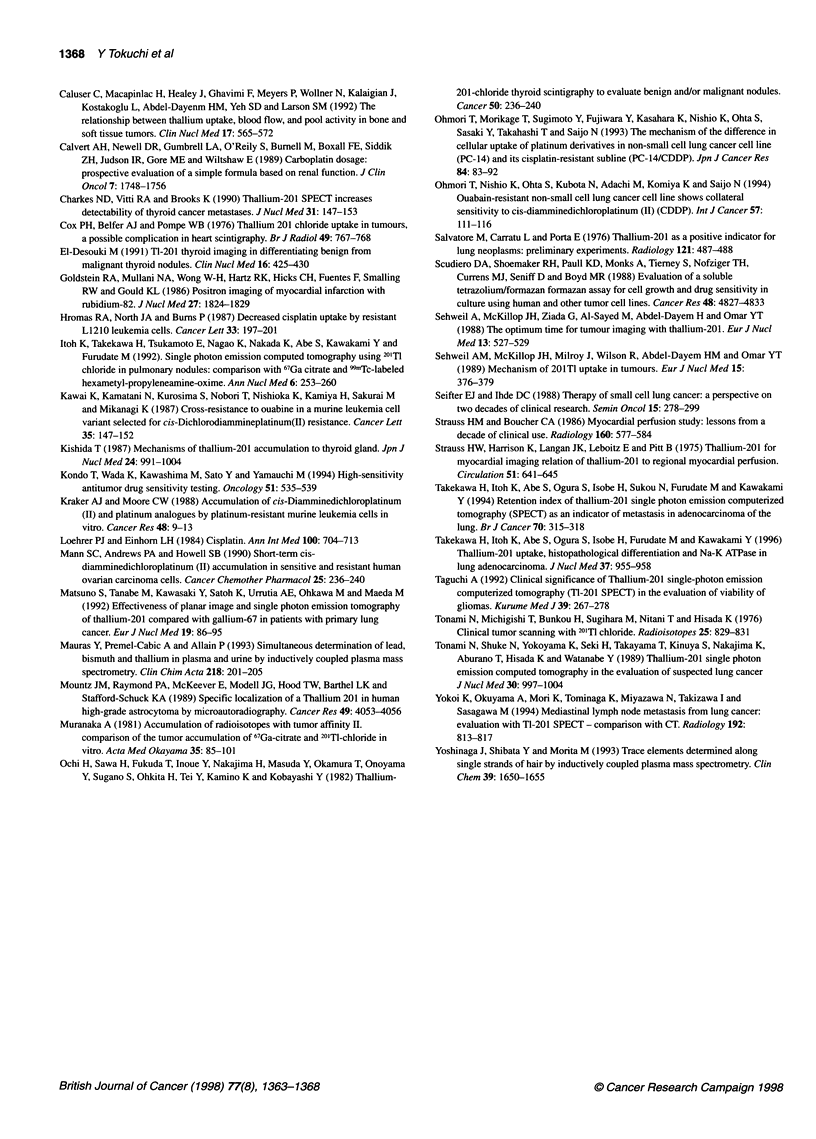# Predicting chemotherapeutic response to small-cell lung cancer of platinum compounds by thallium-201 single-photon emission computerized tomography.

**DOI:** 10.1038/bjc.1998.227

**Published:** 1998-04

**Authors:** Y. Tokuchi, H. Isobe, H. Takekawa, T. Hanada, T. Ishida, S. Ogura, K. Itoh, M. Furudate, K. Saito, Y. Kawakami

**Affiliations:** First Department of Medicine, School of Medicine, Hokkaido University, Nishi, Sapporo, Japan.

## Abstract

Thallium-201 single-photon emission computerized tomography (SPECT) was used to clarify the relationship between 201Tl uptake and the response in chemotherapy to platinum compounds in 21 patients with small-cell lung cancer. 201Tl-SPECT scans were obtained twice: at 15 min (early scan) and 120 min (delayed scan) after an intravenous injection of 111 MBq (3 mCi) of thallium-201 chloride. We obtained the uptake ratio from each scan and calculated the retention index:uptake ratio = region of interest uptake/contralateral normal lung uptake; retention index = (delayed ratio - early ratio)/early ratio. After 201Tl scintigraphy, 12 patients received chemotherapy consisting of platinum compounds and nine were treated with chemoradiation. Among patients receiving only chemotherapy, the retention index correlated with the responses to chemotherapy. In an in vitro study, ouabain, an inhibitor of the Na,K-ATPase pump, reduced sensitivity to cisplatin and inhibited intracellular thallium uptake in the small-cell lung cancer cell line. These studies suggest that 201Tl-SPECT is a useful indicator of response to chemotherapy with platinum compounds in small-cell lung cancer, and that Na,K-ATPase is commonly involved in transporting both thallium and platinum compounds into cancer cells.


British Journal of Cancer (1998) 77(8), 1363–1368
© 1998 Cancer Research Campaign

### Predicting chemotherapeutic response to small-cell lung cancer of platinum compounds by thallium-201 single-photon emission computerized tomography

Y Tokuchi[1], H Isobe[2], H Takekawa[1], T Hanada[1], T Ishida[1], S Ogura[1], K Itoh[3], M Furudate[3], K Saito[4] and Y Kawakami[1]

[1]First Department of Medicine, School of Medicine, Hokkaido University, Kita 15, Nishi 7, Sapporo, 060, Japan; [2]Department of Pulmonary Diseases and Clinical Research Institute, Sapporo National Hospital, Sapporo, Japan; [3]Department of Nuclear Medicine, School of Medicine, Hokkaido University, Kita 15, Nishi 7, Sapporo, 060, Japan; [4]Department of Hygiene and Preventive Medicine, School of Medicine, Hokkaido University, Kita 15, Nishi 7, Sapporo, 060, Japan

**Summary** Thallium-201 single-photon emission computerized tomography (SPECT) was used to clarify the relationship between $^{201}$Tl uptake and the response in chemotherapy to platinum compounds in 21 patients with small-cell lung cancer. $^{201}$Tl-SPECT scans were obtained twice: at 15 min (early scan) and 120 min (delayed scan) after an intravenous injection of 111 MBq (3 mCi) of thallium-201 chloride. We obtained the uptake ratio from each scan and calculated the retention index:uptake ratio = region of interest uptake / contralateral normal lung uptake; retention index = (delayed ratio – early ratio)/early ratio. After $^{201}$Tl scintigraphy, 12 patients received chemotherapy consisting of platinum compounds and nine were treated with chemoradiation. Among patients receiving only chemotherapy, the retention index correlated with the responses to chemotherapy. In an in vitro study, ouabain, an inhibitor of the Na,K-ATPase pump, reduced sensitivity to cisplatin and inhibited intracellular thallium uptake in the small-cell lung cancer cell line. These studies suggest that $^{201}$Tl-SPECT is a useful indicator of response to chemotherapy with platinum compounds in small-cell lung cancer, and that Na,K-ATPase is commonly involved in transporting both thallium and platinum compounds into cancer cells.

**Keywords:** $^{201}$Tl-SPECT; chemotherapy; inductively coupled plasma mass spectrometry; cisplatin; Na,K-ATPase

Cisplatin (CDDP) is a useful anti-cancer agent, particularly when used in the treatment of human ovarian, testicular, bladder and small-cell lung cancer (Loehrer et al, 1984; Ohmori et al, 1993). Many studies on cisplatin resistance mechanisms have revealed that the decline in intracellular accumulation of cisplatin is important in carcinoma cell lines (Hromas et al, 1987; Kraker and Moore, 1988; Andrews and Howell, 1990; Mann et al, 1990). Cisplatin accumulation in these cells is reported to be regulated by an alteration in their Na,K-ATPase levels (Kawai et al, 1987; Andrews et al, 1991; Ohmori et al, 1993). Thus, the response to chemotherapy with cisplatin might depend on alterations of Na,K-ATPase.

Thallium-201 ($^{201}$Tl) scintigraphy is now used to diagnose myocardial infarction (Strauss et al, 1975), myocardial ischaemia (Strauss and Boucher, 1986) and thyroid tumour (Bleichrodt et al, 1987; Charkes et al, 1990). Recently, $^{201}$Tl single-photon emission computerized tomography (SPECT) was reported as being used to detect lung lesion (Tonami et al, 1989), and it is reportedly superior to gallium scintigraphy in detecting lung cancer (Itoh et al, 1992; Matsuno et al, 1992). Some studies demonstrated that Tl accumulation in $^{201}$Tl scintigraphy is closely related to the Na,K-ATPase levels in malignant tumours (Britten and Blank, 1968; Muranaka, 1981; Kishida, 1987; Sehweil et al, 1989). And we reported that the delayed ratio is related to low levels of Na,K-ATPase activity (Takekawa et al, 1996).

*Received 30 January 1997*
*Revised 29 July 1997*
*Accepted 29 September 1997*

*Correspondence to:* Y Tokuchi

We hypothesized that the degree of Tl uptake to tumour in $^{201}$Tl scintigraphy is associated with the response to chemotherapy with platinum compounds that have uptake mechanisms similar to Tl. To clarify the relationship between Tl uptake and the response to chemotherapy with platinum compounds, we examined patients with small-cell lung cancers. Furthermore, we studied in vitro the effect of pretreatment with ouabain, a Na,K-ATPase inhibitor, on the amount of intracellular Tl uptake and the change in sensitivity to CDDP to clarify the relationship between intracellular thallium uptake and sensitivity to cisplatin in the small-cell lung cancer cell line (Ohmori et al, 1993, 1994).

A new method for the assay of intracellular Tl accumulation was used: inductively coupled plasma mass spectrometry (ICP-MS). ICP-MS is analytical technique with high sensitivity for the determination of trace elements in biological samples. The ICP-MS has several advantages for trace elements determination: simultaneous multielements determination; ultrasensitive detection; wide dynamic range (Mauras et al, 1993; Yoshinaga et al, 1993).

### MATERIALS AND METHODS

### Patients

We studied 25 new patients who had small-cell lung carcinomas: they were examined by $^{201}$Tl-SPECT in our hospital from 1991 to 1996. They had received no previous chemotherapy or radiation therapy. Diagnosis was made by the cytology of the endoscopic sampling method (catheter biopsy, bronchoalveolar lavage) and/or histopathology of endoscopic forceps biopsy. We excluded four patients out of the 25: one patient whose primary nodule was too small (less than 15 mm in minor axis) for the sensitivity of

$^{201}$Tl-SPECT in lung cancers (Tonami et al, 1989), one patient who suddenly died because of haemoptysis and two patients who were given reduced platinum compound doses because of their general physical condition. Patients were considered to have limited-stage disease (LD) if detectable cancer was limited to one lung, the mediastinum and the ipsilateral supraclavicular lymph nodes. Extensive-stage disease (ED), then, is defined as any stage of lung cancer more advanced than the limited-stage disease. Each patient gave informed consent.

### $^{201}$Tl-SPECT scanning

Before chemotherapy or chemoradiation therapy, $^{201}$Tl-SPECT scans were obtained twice: first at 15 min (early scan) and then at 120 min (delayed scan) after an intravenous injection of 111 MBq (3 mCi) of thallium-201 chloride. Sehweil et al (1988) reported that $^{201}$Tl uptake occurs rapidly in tumours, with peak values obtained 10–15 min post-injection in most cases, and there were no significant changes in tumour–background ratio between the 1 h post-injection image and 4 h post-injection image. Furthermore, we have previously reported that $^{201}$Tl-SPECT scanning at 120 min is associated with the Na,K-ATPase level of the tumour (Takekawa et al, 1996). Thus, 15 min and 120 min were thought to be suitable times to take images in $^{201}$Tl-SPECT. A gamma-camera (GE-Maxi 400 AT/C) equipped with a parallel-hole collimator was interfaced with a detection computer (Starcom II). Focusing on the chest, the detector was rotated in stages of approximately 6° for a total of 360°. Transaxial images were reconstructed with a Hanning pre-filter and a Ramp post-filter. Coronal and sagittal section images were assembled from transaxial images (Itoh et al, 1992; Takekawa et al, 1994; Takekawa et al, 1996). Without prior knowledge of the cytological or pathological findings, all of the images were interpreted for the presence or absence of abnormal accumulations at a conference of nuclear medicine specialists. When the $^{201}$Tl-SPECT scan showed an abnormal uptake in a lesion, regions of interest (ROIs) were determined and established in both the area with abnormal radioactivity and the contralateral normal lung on the coronal sections of both the early and delayed scans. The mean voxel counts for the ROIs were measured and the uptake ratios of the lesion to the contralateral normal lung were calculated for both the early and delayed scans. We calculated the retention index (Tonami et al, 1989; Takekawa et al, 1994) to quantitatively evaluate the degree of $^{201}$Tl retention in the nodule:retention index = (delayed ratio – early ratio)/early ratio.

### Treatment

Protocol was decided by lung cancer treatment committees according to the patient's performance status, renal function, respiratory function, cardiac condition, liver function and peripheral blood cell counts. In general, treatment was as powerful as the patients could bear. After Tl scintigraphy, patients received combination chemotherapy: CDDP 80 mg $m^{-2}$ on day 1 and etoposide 100 mg $m^{-2}$ on days 1, 3 and 5 or carboplatin (CBDCA) 300–600 mg on day 1 and etoposide 100 mg $m^{-2}$ on days 1, 3 and 5. Carboplatin dosage was determined according to Calvert's formula (Calvert et al, 1989).

Thoracic radiation therapy consisting of 50–60 Gy in 20–24 fractions over 5–6 weeks was simultaneously used with chemotherapy in LD patients who had good respiratory function, good performance status and no interstitial pneumonitis on chest radiography or computerized tomography. When the ED patients had brain metastasis or painful bone metastasis, metastatic sites were simultaneously treated with radiation therapy. The chemotherapy group was defined as those treated with chemotherapy only, and the chemoradiation group was defined as the patients treated with both chemotherapy and radiation therapy at any site. Chemotherapy was repeated for at least two courses; in cases of partial response or complete response, as mentioned below, chemotherapy was repeated every 4 weeks until the time it was determined the tumour would not decrease in volume. At that point, in cases of no change or progressive disease, we chose palliative therapy for better quality of life of the patient.

### Response criteria

In chemotherapy, response evaluation was performed using traditional criteria: a complete response (CR) was defined as the disappearance of all evidence of disease for 4 weeks; a partial response (PR) was defined as a 50% or greater reduction in the sum of the product of the two greatest perpendicular diameters of all measurable lesions for more than 4 weeks, without the appearance of new lesions or the progression of any lesion; no change (NC) was defined as a less than 50% decrease or a less than 25% increase in the sum of the product of the two greatest perpendicular diameters of lesions with no new lesions; and progressive disease (PD) was defined as a 25% or more increase in the size of one or more lesions, or the appearance of new lesions. A good response was defined as CR or PR, and the response rate (%) was defined as good response patients/all patients × 100.

### Drugs and chemicals

Thallium was purchased from Wako Chemical (Osaka, Japan); CDDP and ouabain were purchased from Sigma Chemical (St Louis, MO, USA); RPMI-1640 was purchased from Nissui Pharmaceutical (Tokyo, Japan).

### Cell line

Human small-cell lung cancer cell line PC-6 was obtained from the Japanese Cancer Research Resources Bank (Tokyo, Japan). This cell line was cultivated in tissue culture flasks (Falcon) in RPMI-1640 medium with 10% fetal bovine serum, 100 units $ml^{-1}$ penicillin and 100 μg $ml^{-1}$ streptomycin.

### Thallium accumulation test

PC-6 cells ($1 \times 10^6$) were preincubated in 10 cm tissue culture plates (Falcon 3003) overnight. Ouabain (200 μM) was added to the cells and they were incubated for 1 h (subsequently, PC-6/O), after which the medium was fully vacuumed. An aliquot (10 ml) of RPMI-1640 containing 10 p.p.m. thallium was added to cells for 10 min, followed by two washes with phosphate-buffered saline (PBS). A 1-ml sample of 0.9% sodium chloride was added, and cells were harvested by scraping and then transferred to plastic tubes. Next, 1 ml of 100% nitric acid was added to the tubes and the solutions were taken up into Teflon resolution vessels (Flon Industry, Tokyo, Japan). The vessels were placed in a vacuum oven and heated for 2 h. After cooling, the samples were diluted to 10 ml with Millipore MiliQ water.

For control PC-6, the same procedures were used, but no ouabain was added.

 *© Cancer Research Campaign 1998*

**Table 1** Characteristics of chemotherapy patients and responses to therapy

| Number | Age (years) | Sex | Retention index | Stage | Response | Regimen |
|---|---|---|---|---|---|---|
| 1 | 61 | M | 0.39 | ED | CR | CDDP, etoposide |
| 2 | 64 | M | 0.14 | LD | CR | CDDP, etoposide |
| 3 | 72 | F | 0.28 | ED | PR | CBDCA, etoposide |
| 4 | 69 | M | 0.06 | ED | PR | CBDCA, etoposide |
| 5 | 72 | M | 0.21 | LD | PR | CDDP, etoposide |
| 6 | 58 | M | –0.05 | ED | NC | CDDP, etoposide |
| 7 | 65 | M | 0.00 | ED | NC | CDDP, etoposide |
| 8 | 78 | M | 0.11 | LD | NC | CDDP, etoposide |
| 9 | 82 | M | –0.16 | ED | NC | CBDCA, etoposide |
| 10 | 63 | M | 0.12 | ED | PD | CDDP, etoposide |
| 11 | 79 | M | 0.00 | LD | PD | CBDCA, etoposide |
| 12 | 66 | M | 0.03 | ED | PD | CDDP, etoposide |

ED, extensive disease; LD, limited disease.

### Instrumentation and samples

The inductively coupled plasma mass spectrometry (ICP-MS) apparatus used was an SPQ6500 (Seiko Instruments, Shizuoka, Japan), with a nebulizer and a spray chamber made of borosilicate glass. The digested samples were nebulized into ICP-MS without further dilution. Quantification of all samples was performed using external standard solutions made up of 1% nitric acid. The standard solutions used 10, 50 and 100 p.p.b. thallium and, as a control solution, 0.1% nitric acid. Each experiment was repeated three times.

### Drug sensitivity test

Chemosensitivity of the cells to CDDP was determined using the XTT assay, as previously reported (Scudiero et al, 1988; Kondo et al, 1994). After preincubation at 37°C overnight, the 200 μM ouabain was added to PC-6 (now, PC-6/O), incubated for 1 h at 37°C and washed twice with PBS. Various concentrations of the CDDP were added to PC-6 and PC-6/O, and all cells were incubated for 1 h and washed twice with PBS. Units of 1000 cells were seeded into 96-well microplates and incubated at 37°C for 96 h. After incubation, 50 μg of 2,3-bis (2-methoxy-4-nitro-5-sulphophenyl) [(phenyl-amino) carbonyl]-$^2$H-tetrazolium hydroxide (XTT, Sigma, St Louis, MO, USA) with 0.38 μg of phenazine methosulphate (PMS, Sigma) were added to each well and incubated at 37°C for 4 h. The plates were agitated on a plate shaker for 5 min, and the absorbance was measured at 450 nm using an ELISA reader. The drug concentration producing 50% inhibition of growth ($IC_{50}$) was determined from a standard concentration–response curve. Each experiment was repeated three times.

### Statistics

In clinical study, between-group comparisons were carried out using the Mann–Whitney *U*-test. Differences were considered significant when the *P*-value was less than 0.05.

In an in vitro study, comparisons between means of thallium accumulation and $IC_{50}$ on CDDP sensitivity test were performed using Student's *t*-test. Differences were considered significant when the *P*-value was less than 0.05.

### RESULTS

The characteristics of the 12 patients treated with only chemotherapy are listed in Table 1, and the characteristics of the nine patients treated with chemoradiation therapy are listed in Table 2. The overall response rate was 62%; CR rate was 33% of the 21 patients.

Figure 1 shows that the retention indexes for the PR and the CR groups were significantly higher than those of the PD and the NC groups among patients treated only with chemotherapy ($P = 0.012$). Except for CBDCA patients, the difference is clear too ($P = 0.025$). This demonstrated that the retention index was a reliable indicator for chemotherapy among patients with small-cell lung carcinomas. However, if we consider all data in both Tables 1 and 2, the difference between them is not clear ($P = 0.075$): combining radiation therapy with chemotherapy might overcome the relationship between the retention index and the effect of chemotherapy. Considering only the patients who did not receive thoracic radiation (numbers 2, 4, 6, 7 in Table 2) in Table 1, the difference was statistically consistent ($P = 0.043$).

In contrast, the early ratio and the delayed ratio showed no association with chemotherapeutic response among these patients (data not shown).

Figure 2 shows the standard curve for thallium determination by ICP-MS. The correlation coefficient of the regression line was 0.999. ICP-MS is an analytic technique with high sensitivity, and the standard curve has a wide linear range in measuring Tl concentration, as previously reported (Yoshinaga, et al, 1993; Mauras et al, 1993).

Intracellular thallium uptake is demonstrated in PC-6 and PC-6/O, as ouabain-treated PC-6 cells, in Figure 3. The level of intracellular thallium in PC-6/O is clearly lower than in PC-6 after contact with thallium. We obtained the same result with successive experiments: Ouabain reduced intracellular thallium uptake in the PC-6 cell line.

Figure 4 shows the result of the cisplatin sensitivity test. PC-6/O was pretreated with ouabain and showed less response to CDDP than did PC-6. The $IC_{50}$ for PC-6 was 3.1 μM; for PC-6/O, 5.1 μM. There is a significant difference between them ($P < 0.05$), demonstrating that ouabaine reduced sensitivity to CDDP in the PC-6 cell line.

© Cancer Research Campaign 1998 

**Table 2** Characterisics of patients receiving chemoradiation therapy and responses to therapy

| Number | Age (years) | Sex | Retention index | Stage | Response | Regimen |
|---|---|---|---|---|---|---|
| 1 | 72 | M | 0.00 | LD | CR | CDDP, etoposide |
| 2 | 64 | M | 0.03 | ED | CR | CDDP, etoposide |
| 3 | 68 | F | 0.22 | LD | CR | CDDP, etoposide |
| 4 | 74 | F | 0.07 | ED | CR | CDDP, etoposide |
| 5 | 74 | M | 0.01 | LD | CR | CBDCA, etoposide |
| 6 | 56 | M | 0.00 | ED | PR | CDDP, etoposide |
| 7 | 75 | M | 0.20 | LD | PR | CBDCA, etoposide |
| 8 | 60 | M | 0.08 | ED | PR | CDDP, etoposide |
| 9[a] | 65 | M | 0.18 | ED | PD | CBDCA, etoposide |

LD, limited disease; ED, extensive disease. [a]Patient number 9 was simultaneously given treatment with thoracic radiation because the primary tumour had painfully invaded the chest wall.

**Figure 1** Retention index and responses to chemotherapy among patients with small-cell carcinomas. Points and bars are mean and standard deviations (s.d.)

**Figure 3** Amount of cellular Tl in PC-6 and PC-6/O. ○, PC-6; □, PC-6/O. Each data point and bar are the mean and s.d. of three experiments

**Figure 2** Calibration curve on measuring Tl with ICP-MS

### DISCUSSION

Thallium-201 chloride has been described as a positive indicator for lung cancer (Cox et al, 1976; Salvatore et al, 1976; Tonami et al, 1976). SPECT provides a significant improvement, with respect to the radiopharmaceutical distribution in the body in three dimensions and the ability to extract true quantitative values from structures deep within the body (Matsuno et al, 1992; Yokoi et al, 1994). $^{201}$Tl-SPECT has been reported to visualize lung cancers as small as 1.5 × 1.0 cm (Tonami, et al, 1989), and 1.0 × 1.0 cm (Matsuno et al, 1992). It is possible to diagnose a lung tumour larger than 15 mm in diameter as benign or malignant (Tonami et al, 1989).

The accumulation patterns of $^{201}$Tl on early and delayed scans differ between benign and malignant lung and thyroid tumours (Ochi et al, 1982; Tonami et al, 1989; El-desouki 1991). In benign tumours, $^{201}$Tl shows either negative or reduced accumulation on the delayed scan. Malignant tumours, on the other hand, clearly

© Cancer Research Campaign 1998

**Figure 4** CDDP sensitivity tests. O, PC-6; □, PC-6/O. Each data point and bar are the mean and s.d. of three experiments show accumulation of $^{201}$Tl on both the early and the delayed scans. Retention of $^{201}$Tl on the delayed scan is strongly suggestive of malignancy. In rats, the $^{201}$Tl accumulation revealed in inflammatory lesions decreased with time, but $^{201}$Tl washout from malignant tumours tended to be delayed (Ando et al, 1987).

In $^{201}$Tl scintigraphy, the early ratio in a tumour reflects vascularity (Taguchi 1992) and blood pooling (Caluser et al, 1992), whereas the delayed ratio reflects the cell's ability to increase Tl/Na,K-ATP levels (Ando et al, 1988; Caluser et al, 1992; Takekawa et al, 1996) as well as the viability of tumour cells (Mountz, et al 1989; Sehweil et al, 1989). The retention index was defined thus: retention index = (delayed ratio – early ratio)/early ratio. Defining the early ratio as vascularity and the delayed ratio as Na,K-ATPase, this formula would mean that Na,K-ATPase varied with vascularity. Therefore, the retention index would be a stronger indicator of active transportation by Na,K-ATPase into cancer cells in the body than would the delayed ratio by itself.

In an in vitro study, influx of thallium into malignant cells is regulated by its active transportation by Na,K-ATPase (Muranaka, 1981; Kishida, 1987; Sehweil et al, 1989). Since we showed that the Na,K-ATPase inhibitor ouabain blocks intracellular thallium uptake in the PC-6 cell line, Na,K-ATPase must play a vital role in intracellular thallium uptake.

In this study, the retention index of $^{201}$Tl-SPECT was associated with the response to chemotherapy with platinum compounds in small-cell lung carcinomas. Therefore, the retention index might be an indicator of response to chemotherapy with platinum compounds in small-cell lung carcinomas.

Although chemotherapy with platinum compounds has potency in small-cell lung cancers, especially in combination with etoposide (Seifter and Ihde, 1988), the responses vary from complete recovery to progression of the disease. Cisplatin accumulation in these cells is probably regulated by an alteration in their Na,K-ATPase levels (Kishida, 1987; Andrews et al, 1991; Ohmori et al, 1993). Many cisplatin-resistant cell lines show cross-resistance to carboplatin (Kraker et al, 1988; Ohmori et al, 1993). Ohmori reported that PC-14/CDDP, a cisplatin-resistant non-small-cell lung cancer cell line, showed 3.5-fold resistance to carboplatin, and that the accumulation of cisplatin and carboplatin decreased to 23% and 27%. In addition, ouabain inhibited 60% of CDDP accumulation in PC-14, whereas the same dose of ouabain did not affect CDDP accumulation in PC-14/CDDP (Ohmori et al, 1993). Thus, carboplatin may behave similarly to cisplatin on drug-resistant mechanisms. With or without carboplatin groups, there was no difference in this clinical study in the relationship between $^{201}$Tl accumulation and the chemotherapy effect.

Our in vitro study suggested that the sensitivity to CDDP might be associated with the intracellular uptake of thallium by alterations in Na,K-ATPase in the small-cell lung cancer cell line. In addition, the accumulation in cancer cells of thallium and platinum compounds might be regulated by a common mechanism: active transportation by Na,K-ATPase. Together, these indicated that the accumulation shown in the tumour in $^{201}$Tl-SPECT correlated with that of the platinum compounds. To our knowledge, this is the first report demonstrating a significant relationship between the uptake ratios of $^{201}$Tl-SPECT and the prediction of chemoresponse to platinum compounds.

Because we used the standard treatment for small-cell lung cancer patients in this clinical study, i.e. combination chemotherapy with platinum compounds and etoposide, we cannot disregard etoposide effects. Therefore, other studies might be needed to determine the effects of etoposide.

Recently rubidium-82 ($^{82}$Rb) and positron emission tomography (PET) have been used to diagnose myocardial infarction (Goldstein et al, 1986). Because $^{82}$Rb is taken up into cells by Na,K-ATPase in the same way as $^{201}$Tl – and as PET gives more accurate images than $^{201}$Tl SPECT – a follow-up study using $^{82}$Rb and PET might clarify the relationship between uptake and chemotherapeutic response.

The benefit of $^{201}$Tl-SPECT is its non-invasiveness: $^{201}$Tl-SPECT can give a safe, reliable prediction on a patient's response to chemotherapy. If we can predict the response, we can avoid ineffective chemotherapy and unfavourable toxicity, and choose more suitable drugs for lung cancer patients.

In conclusion, $^{201}$Tl-SPECT could be a useful indicator of response to chemotherapy with platinum compounds in lung cancers.

### ACKNOWLEDGEMENTS

The authors would like to thank Nuclear Medicine Service Fellows and Lung Cancer Treatment Committees in Hokkaido University for referring patients during this study.

### REFERENCES

Ando A, Ando I, Katayama M, Sanada S, Hiraki T, Mori H, Tonami N and Hisada K (1988) Biodistributions of radioactive alkaline metals in tumor bearing animals: comparison with $^{201}$Tl. *Eur J Nucl Med* **14**: 352–357

Andrews PA and Howell SB (1990). Cellular pharmacology of cisplatin: perspectives on mechanisms of acquired resistance. *Cancer Cells* **2**: 35–43

Andrews PA, Mann SC, Huynh HH and Albright K (1991) Role of the $Na^+,K^+$-adenosine triphosphatase in the accumulation of *cis*-Diamminedichloroplatinum (II) in human ovarian carcinoma cells. *Cancer Res* **51**: 3677–3681

Bleichrodt RP, Vermey A, Apiers D and De Langen ZJ (1987) Early and delayed thallium 201 imaging. *Cancer* **60**: 2621–2623

Britten JS and Blank M (1968) Thallium activation of the ($Na^+$-$K^+$)-activated ATPase of rabbit kidney. *Biochem Biophys Acta* **159**: 160–166

© Cancer Research Campaign 1998 

Caluser C, Macapinlac H, Healey J, Ghavimi F, Meyers P, Wollner N, Kalaigian J, Kostakoglu L, Abdel-Dayenm HM, Yeh SD and Larson SM (1992) The relationship between thallium uptake, blood flow, and pool activity in bone and soft tissue tumors. *Clin Nucl Med* **17**: 565–572

Calvert AH, Newell DR, Gumbrell LA, O'Reily S, Burnell M, Boxall FE, Siddik ZH, Judson IR, Gore ME and Wiltshaw E (1989) Carboplatin dosage: prospective evaluation of a simple formula based on renal function. *J Clin Oncol* **7**: 1748–1756

Charkes ND, Vitti RA and Brooks K (1990) Thallium-201 SPECT increases detectability of thyroid cancer metastases. *J Nucl Med* **31**: 147–153

Cox PH, Belfer AJ and Pompe WB (1976) Thallium 201 chloride uptake in tumours, a possible complication in heart scintigraphy. *Br J Radiol* **49**: 767–768

El-Desouki M (1991) Tl-201 thyroid imaging in differentiating benign from malignant thyroid nodules. *Clin Nucl Med* **16**: 425–430

Goldstein RA, Mullani NA, Wong W-H, Hartz RK, Hicks CH, Fuentes F, Smalling RW and Gould KL (1986) Positron imaging of myocardial infarction with rubidium-82. *J Nucl Med* **27**: 1824–1829

Hromas RA, North JA and Burns P (1987) Decreased cisplatin uptake by resistant L1210 leukemia cells. *Cancer Lett* **33**: 197–201

Itoh K, Takekawa H, Tsukamoto E, Nagao K, Nakada K, Abe S, Kawakami Y and Furudate M (1992). Single photon emission computed tomography using $^{201}$Tl chloride in pulmonary nodules: comparison with $^{67}$Ga citrate and $^{99m}$Tc-labeled hexametyl-propyleneamine-oxime. *Ann Nucl Med* **6**: 253–260

Kawai K, Kamatani N, Kurosima S, Nobori T, Nishioka K, Kamiya H, Sakurai M and Mikanagi K (1987) Cross-resistance to ouabine in a murine leukemia cell variant selected for *cis*-Dichlorodiammineplatinum(II) resistance. *Cancer Lett* **35**: 147–152

Kishida T (1987) Mechanisms of thallium-201 accumulation to thyroid gland. *Jpn J Nucl Med* **24**: 991–1004

Kondo T, Wada K, Kawashima M, Sato Y and Yamauchi M (1994) High-sensitivity antitumor drug sensitivity testing. *Oncology* **51**: 535–539

Kraker AJ and Moore CW (1988) Accumulation of *cis*-Diamminedichloroplatinum (II) and platinum analogues by platinum-resistant murine leukemia cells in vitro. *Cancer Res* **48**: 9–13

Loehrer PJ and Einhorn LH (1984) Cisplatin. *Ann Int Med* **100**: 704–713

Mann SC, Andrews PA and Howell SB (1990) Short-term cis-diamminedichloroplatinum (II) accumulation in sensitive and resistant human ovarian carcinoma cells. *Cancer Chemother Pharmacol* **25**: 236–240

Matsuno S, Tanabe M, Kawasaki Y, Satoh K, Urrutia AE, Ohkawa M and Maeda M (1992) Effectiveness of planar image and single photon emission tomography of thallium-201 compared with gallium-67 in patients with primary lung cancer. *Eur J Nucl Med* **19**: 86–95

Mauras Y, Premel-Cabic A and Allain P (1993) Simultaneous determination of lead, bismuth and thallium in plasma and urine by inductively coupled plasma mass spectrometry. *Clin Chim Acta* **218**: 201–205

Mountz JM, Raymond PA, McKeever E, Modell JG, Hood TW, Barthel LK and Stafford-Schuck KA (1989) Specific localization of a Thallium 201 in human high-grade astrocytoma by microautoradiography. *Cancer Res* **49**: 4053–4056

Muranaka A (1981) Accumulation of radioisotopes with tumor affinity II. comparison of the tumor accumulation of $^{67}$Ga-citrate and $^{201}$Tl-chloride in vitro. *Acta Med Okayama* **35**: 85–101

Ochi H, Sawa H, Fukuda T, Inoue Y, Nakajima H, Masuda Y, Okamura T, Onoyama Y, Sugano S, Ohkita H, Tei Y, Kamino K and Kobayashi Y (1982) Thallium-201-chloride thyroid scintigraphy to evaluate benign and/or malignant nodules. *Cancer* **50**: 236–240

Ohmori T, Morikage T, Sugimoto Y, Fujiwara Y, Kasahara K, Nishio K, Ohta S, Sasaki Y, Takahashi T and Saijo N (1993) The mechanism of the difference in cellular uptake of platinum derivatives in non-small cell lung cancer cell line (PC-14) and its cisplatin-resistant subline (PC-14/CDDP). *Jpn J Cancer Res* **84**: 83–92

Ohmori T, Nishio K, Ohta S, Kubota N, Adachi M, Komiya K and Saijo N (1994) Ouabain-resistant non-small cell lung cancer cell line shows collateral sensitivity to cis-diamminedichloroplatinum (II) (CDDP). *Int J Cancer* **57**: 111–116

Salvatore M, Carratu L and Porta E (1976) Thallium-201 as a positive indicator for lung neoplasms: preliminary experiments. *Radiology* **121**: 487–488

Scudiero DA, Shoemaker RH, Paull KD, Monks A, Tierney S, Nofziger TH, Currens MJ, Seniff D and Boyd MR (1988) Evaluation of a soluble tetrazolium/formazan formazan assay for cell growth and drug sensitivity in culture using human and other tumor cell lines. *Cancer Res* **48**: 4827–4833

Sehweil A, McKillop JH, Ziada G, Al-Sayed M, Abdel-Dayem H and Omar YT (1988) The optimum time for tumour imaging with thallium-201. *Eur J Nucl Med* **13**: 527–529

Sehweil AM, McKillop JH, Milroy J, Wilson R, Abdel-Dayem HM and Omar YT (1989) Mechanism of 201Tl uptake in tumours. *Eur J Nucl Med* **15**: 376–379

Seifter EJ and Ihde DC (1988) Therapy of small cell lung cancer: a perspective on two decades of clinical research. *Semin Oncol* **15**: 278–299

Strauss HM and Boucher CA (1986) Myocardial perfusion study: lessons from a decade of clinical use. *Radiology* **160**: 577–584

Strauss HW, Harrison K, Langan JK, Leboitz E and Pitt B (1975) Thallium-201 for myocardial imaging relation of thallium-201 to regional myocardial perfusion. *Circulation* **51**: 641–645

Takekawa H, Itoh K, Abe S, Ogura S, Isobe H, Sukou N, Furudate M and Kawakami Y (1994) Retention index of thallium-201 single photon emission computerized tomography (SPECT) as an indicator of metastasis in adenocarcinoma of the lung. *Br J Cancer* **70**: 315–318

Takekawa H, Itoh K, Abe S, Ogura S, Isobe H, Furudate M and Kawakami Y (1996) Thallium-201 uptake, histopathological differentiation and Na-K ATPase in lung adenocarcinoma. *J Nucl Med* **37**: 955–958

Taguchi A (1992) Clinical significance of Thallium-201 single-photon emission computerized tomography (Tl-201 SPECT) in the evaluation of viability of gliomas. *Kurume Med J* **39**: 267–278

Tonami N, Michigishi T, Bunkou H, Sugihara M, Nitani T and Hisada K (1976) Clinical tumor scanning with $^{201}$Tl chloride. *Radioisotopes* **25**: 829–831

Tonami N, Shuke N, Yokoyama K, Seki H, Takayama T, Kinuya S, Nakajima K, Aburano T, Hisada K and Watanabe Y (1989) Thallium-201 single photon emission computed tomography in the evaluation of suspected lung cancer *J Nucl Med* **30**: 997–1004

Yokoi K, Okuyama A, Mori K, Tominaga K, Miyazawa N, Takizawa I and Sasagawa M (1994) Mediastinal lymph node metastasis from lung cancer: evaluation with Tl-201 SPECT – comparison with CT. *Radiology* **192**: 813–817

Yoshinaga J, Shibata Y and Morita M (1993) Trace elements determined along single strands of hair by inductively coupled plasma mass spectrometry. *Clin Chem* **39**: 1650–1655

© *Cancer Research Campaign 1998*